# Selection for feed efficiency using the social effects animal model in growing Duroc pigs: evaluation by simulation

**DOI:** 10.1186/s12711-020-00572-4

**Published:** 2020-09-29

**Authors:** William Herrera-Cáceres, Juan Pablo Sánchez

**Affiliations:** Genetica i Millora Animal, Institut de Recerca i Tecnologia Agroalimentàries (IRTA), Torre Marimon s/n, Caldes de Montbui, Barcelona, 08140 Spain

## Abstract

**Background:**

Traits recorded on animals that are raised in groups can be analysed with the social effects animal model (SAM). For multiple traits, this model specifies the genetic correlation structure more completely than the animal model (AM). Our hypothesis was that by using the SAM for genetic evaluation of average daily gain (ADG) and backfat thickness (BF), a high rate of improvement in feed conversion ratio (FCR) might be achieved, since unfavourable genetic correlations between ADG and BF reported in a Duroc pig line could be partially avoided. We estimated genetic and non-genetic correlations between BF, ADG and FCR on 1144 pigs using Bayesian methods considering the SAM; and responses to selection indexes that combine estimates of indirect (IGE) and direct (DGE) genetic effects for ADG and BF by stochastic simulation.

**Results:**

Estimates of the ratio of the variance of DGE to the phenotypic variance were 0.31, 0.39 and 0.25 and those of the total genetic variance to the phenotypic variance were 0.63, 0.74 and 0.93 for ADG, BF and FCR, respectively. In spite of this, when the SAM was used to generate data and for the genetic evaluations, the average economic response was worse than that obtained when BV predictions from the AM were considered. The achieved economic response was due to a direct reduction in BF and not to an improvement in FCR.

**Conclusions:**

Our results show that although social genetic effects play an important role in the traits studied, their proper consideration in pig breeding programs to improve FCR indirectly is still difficult. The correlations between IGE and DGE that could help to overcome the unfavourable genetic correlations between DGE did not reach sufficiently high magnitudes; also, the genetic parameters estimates from the SAM have large errors. These two factors penalize the average response under the SAM compared to the AM.

## Background

Since feed represents the largest cost of pig production, improving feed efficiency would result in a high economic benefit [1]. For this reason, breeding programs either directly or indirectly focus on improving this trait. Traditionally, indirect improvement in feed efficiency has been achieved by improving growth rate while reducing backfat thickness (BF) [2], but reaching such a joint objective can be difficult because these traits are under antagonist genetic control [3, 4]. The major challenge in breeding programs that address feed efficiency directly is that individual feed intake needs to be recorded. When animals are raised in group pens, this trait can be measured only by using electronic feeders, which assumes a significant investment and greatly increases the production costs in the breeding program. In this context, when animals are housed in groups, feed efficiency traits, as with any other performance trait, can be affected by social interactions; a concept that was first introduced by Griffing 1976 [5]. The social effects animal model (SAM) assumes that a trait is controlled by two genetic effects: the direct genetic effect (DGE) due to genes that have a direct effect on the performance of the animal itself, and indirect genetic effect (IGE) due to genes of its pen mates that act on the animal that is recorded [6]. By using the SAM, Nielsen et al. [7] and Ragab et al. [8] reported a better predictive ability for growth traits in pigs compared to that of a model without IGE.

In this study, our aim was to explore whether the expected response in feed conversion ratio (FCR), as a result of indirect selection for average daily gain (ADG) and BF when using the SAM for genetic evaluation, is improved compared to that achieved when using the traditional animal model (AM). In this analysis, we accounted for the uncertainty regarding the genetic and non-genetic parameters that are required to conduct the evaluation of the selection candidates. For the Duroc population considered in this study, we have already reported genetic correlation estimates between performances (ADG and BF) and feed efficiency traits (FCR) using the AM and SAM [9]. Based on these estimates, some advantages for the use of the SAM would be expected for indirect genetic improvement of FCR because it enables different selection pressures to be placed on direct versus indirect genetic effects for traits with unfavourable genetic correlations, thereby partially avoiding the estimated antagonist genetic relationship under the traditional animal model.

## Methods

### Animals

Performance during the growing period was recorded on 1144 Duroc pigs in 10 batches from 2007 to 2017 at the Center of Porcine Evaluation (Monells, Girona, Spain) using IVOG feeding stations (Insentec, Markenesse, The Netherland) with one single-space electronic feeder per pen. Herrera-Cáceres et al. [9] described the Duroc line used in this study in detail. The population under study was housed in 97 pens and pen size varied from 7 to 15 animals, with an average pen size of 12.0 ± 1.5 animals. Age of the animals at the beginning and the end of the fattening period was 70 ± 6 days and 177 ± 9 days, respectively.

### Description of traits

For this study, average daily gain (ADG, kg), feed conversion ratio (FCR, kg/kg), and backfat thickness (BF, mm) were computed at 180 days of age, as described by Herrera-Cáceres et al. [9]. Basic statistics for these traits are in Table 1.

**Table 1:** Descriptive statistics for growing Duroc pigs

Trait	Abbreviation	Number of observations	Min	Mean	Max	SD
Average daily gain, kg/day	ADG	1144	0.22	0.82	1.07	0.09
Backfat thickness, mm	BF	1144	6.44	18.19	32.74	4.40
Feed conversion ratio, kg/kg	FCR	1144	2.07	2.77	3.89	0.24

### Statistical model

A multi-trait linear model was fitted to ADG, BF, and FCR. The same systematic effects were considered for the three traits: batch, age at the end of the fattening period (covariate), and number of pigs per pen (covariate). In addition, pen, litter, and genetic effects were included. The genetic component of the model was accounted for by fitting direct and indirect genetic effects separately [6], as in the SAM. This three-trait model is represented by the following equation:

1 $$\begin{array}{ccccc}
\left[ {\begin{array}{*{20}{c}}
{\begin{array}{*{20}{c}}
{{{\bf{y}}_1}}\\
{{{\bf{y}}_2}}
\end{array}}\\
{{{\bf{y}}_3}}
\end{array}} \right] =  & \left[ {\begin{array}{*{20}{c}}
{{{\bf{X}}_1}}&0&0\\
0&{{{\bf{X}}_2}}&0\\
0&0&{{{\bf{X}}_3}}
\end{array}} \right]\left[ {\begin{array}{*{20}{c}}
{\begin{array}{*{20}{c}}
{{b_1}}\\
{{b_2}}
\end{array}}\\
{{b_3}}
\end{array}} \right] + \left[ {\begin{array}{*{20}{c}}
{{{\bf{Z}}_{\bf{p}}}_1}&0&0\\
0&{{{\bf{Z}}_{\bf{p}}}_2}&0\\
0&0&{{{\bf{Z}}_{\bf{p}}}_3}
\end{array}} \right]\left[ {\begin{array}{*{20}{c}}
{\begin{array}{*{20}{c}}
{{{\bf{p}}_1}}\\
{{{\bf{p}}_2}}
\end{array}}\\
{{{\bf{p}}_3}}
\end{array}} \right]\\
+ \left[ {\begin{array}{*{20}{c}}
{{{\bf{Z}}_{\bf{l}}}_1}&0&0\\
0&{{{\bf{Z}}_{\bf{l}}}_2}&0\\
0&0&{{{\bf{Z}}_{\bf{l}}}_3}
\end{array}} \right]\left[ {\begin{array}{*{20}{c}}
{\begin{array}{*{20}{c}}
{{{\bf{l}}_1}}\\
{{{\bf{l}}_2}}
\end{array}}\\
{{{\bf{l}}_3}}
\end{array}} \right] + \left[ {\begin{array}{*{20}{c}}
{{{\bf{Z}}_{\bf{d}}}_1}&0&0\\
0&{{{\bf{Z}}_{\bf{d}}}_2}&0\\
0&0&{{{\bf{Z}}_{\bf{d}}}_3}
\end{array}} \right]\left[ {\begin{array}{*{20}{c}}
{\begin{array}{*{20}{c}}
{{{\bf{d}}_1}}\\
{{{\bf{d}}_2}}
\end{array}}\\
{{{\bf{d}}_3}}
\end{array}} \right]\\
+ \left[ {\begin{array}{*{20}{c}}
{{{\bf{Z}}_{\bf{s}}}_1}&0&0\\
0&{{{\bf{Z}}_{\bf{s}}}_2}&0\\
0&0&{{{\bf{Z}}_{\bf{s}}}_3}
\end{array}} \right]\left[ {\begin{array}{*{20}{c}}
{\begin{array}{*{20}{c}}
{{{\bf{s}}_1}}\\
{{{\bf{s}}_2}}
\end{array}}\\
{{{\bf{s}}_3}}
\end{array}} \right] + \left[ {\begin{array}{*{20}{c}}
{\begin{array}{*{20}{c}}
{{{\bf{e}}_1}}\\
{{{\bf{e}}_2}}
\end{array}}\\
{{{\bf{e}}_3}}
\end{array}} \right],
\end{array}$$

where ${\mathbf{y}}_{1}$, ${\mathbf{y}}_{2}$ and ${\mathbf{y}}_{3}$ are the vectors of observations for the first (ADG), second (BF) and third (FCR) trait, respectively, ${\mathbf{b}}$ is a vector of systematic effects with incidence matrix ${\mathbf{X}}$; ${\mathbf{p}}$ is a vector of pen effects with incidence matrix ${\mathbf{Z}}_{{\mathbf{p}}}$; ${\mathbf{l}}$ is a vector of litter effects with incidence matrix ${\mathbf{Z}}_{{\mathbf{l}}}$; ${\mathbf{d}}$ is a vector of direct genetic effects with incidence matrix ${\mathbf{Z}}_{{\mathbf{d}}}$, and ${\mathbf{s}}$ is a vector of indirect genetic effects with incidence matrix ${\mathbf{Z}}_{{\mathbf{s}}}$. The elements of ${\mathbf{Z}}_{\text{s}}$ are 1 for records from animals that share the same pen and 0 otherwise; ${\mathbf{e}}$ is a vector of residuals.

The prior distributions of pen, litter and residual effects were: **p** $$ |{\mathbf{P}}_{0} \sim N\left( {0,{\mathbf{P}}_{0} \otimes {\mathbf{I}}_{\text{p}} } \right) $$, $$ {\mathbf{l}}|{\mathbf{L}}_{0} \sim N\left( {0,{\mathbf{L}}_{0} \otimes {\mathbf{I}}_{{\mathbf{l}}} } \right) $$, and $$ {\mathbf{e}}|{\mathbf{R}}_{0} \sim N\left( {0,{\mathbf{R}}_{0} \otimes {\mathbf{I}}_{{\mathbf{r}}} } \right) $$, where $$ {\mathbf{I}}_{\text{p}} $$,$$ \varvec{ }{\mathbf{I}}_{{\mathbf{l}}} $$ and $$ {\mathbf{I}}_{{\mathbf{r}}} \varvec{ } $$ are identity matrices of appropriate dimensions, $$ {\mathbf{P}}_{0} , $$$$ {\mathbf{L}}_{0} $$ and $$ {\mathbf{R}}_{0} $$ are 3 × 3 covariance matrices for the three traits, and ⊗ denotes the Kronecker product. All factors were assumed to be independent, except direct and indirect genetic effects, for which the assumed prior distribution was: $$ \left[ {\begin{array}{*{20}c} {\mathbf{d}} \\ {\mathbf{s}} \\ \end{array} } \right]|{\mathbf{G}}_{0} \sim {\text{MVN}}\left( {0, {\mathbf{G}}_{0} \otimes {\mathbf{A}}} \right) $$, where $$ {\mathbf{A}} $$ is the numerator relationship matrix between individuals and $$ {\mathbf{G}}_{0} $$ is the covariance matrix, containing the following elements:

$$ {\mathbf{G}}_{0} = \left[ {\begin{array}{*{20}l} {{{\sigma }}_{{{\text{d}}_{1} }}^{2} } \hfill &{{{\sigma }}_{{{\text{d}}_{1} , {\text{d}}_{2} }} } \hfill &{{{\sigma }}_{{{\text{d}}_{1} , {\text{d}}_{3} }} } \hfill &{{{\sigma }}_{{{\text{d}}_{1} , {\text{s}}_{1} }} } \hfill &{{{\sigma }}_{{{\text{d}}_{1} , {\text{s}}_{2} }} } \hfill &{{{\sigma }}_{{{\text{d}}_{1} , {\text{s}}_{3} }} } \hfill \\ {{{\sigma }}_{{{\text{d}}_{1} ,{\text{d}}_{2} }} } \hfill &{{{\sigma }}_{{{\text{d}}_{2} }}^{2} } \hfill &{{{\sigma }}_{{{\text{d}}_{2} , {\text{d}}_{3} }} } \hfill &{{{\sigma }}_{{{\text{d}}_{2} , {\text{s}}_{1} }} } \hfill &{{{\sigma }}_{{   {\text{d}}_{2} , {\text{s}}_{2} }} } \hfill &{{{\sigma }}_{{{\text{d}}_{2} , {\text{s}}_{3} }} } \hfill \\ {{{\sigma }}_{{{\text{d}}_{1} , {\text{d}}_{3} }} } \hfill &{{{\sigma }}_{{{\text{d}}_{2} , {\text{d}}_{3} }} } \hfill &{{{\sigma }}_{{{\text{d}}_{3} }}^{2} } \hfill &{{{\sigma }}_{{{\text{d}}_{3} , {\text{s}}_{1} }} } \hfill &{{{\sigma }}_{{{\text{d}}_{3} , {\text{s}}_{2} }} } \hfill &{{{\sigma }}_{{{\text{d}}_{3} , {\text{s}}_{3} }} } \hfill \\ {{{\sigma }}_{{{\text{d}}_{1} , {\text{s}}_{1} }} } \hfill &{{{\sigma }}_{{{\text{d}}_{2} , {\text{s}}_{1} }} } \hfill &{{{\sigma }}_{{{\text{d}}_{3} , {\text{s}}_{1} }} } \hfill &{{{\sigma }}_{{{\text{s}}_{1} }}^{2} } \hfill &{{{\sigma }}_{{{\text{s}}_{1} , {\text{s}}_{2} }} } \hfill &{{{\sigma }}_{{{\text{s}}_{1} , {\text{s}}_{3} }} } \hfill \\ {{{\sigma }}_{{{\text{d}}_{1} , {\text{s}}_{2} }} } \hfill &{{{\sigma }}_{{{\text{d}}_{2} , {\text{s}}_{2} }} } \hfill &{{{\sigma }}_{{{\text{d}}_{3} , {\text{s}}_{2} }} } \hfill &{{{\sigma }}_{{{\text{s}}_{1} , {\text{s}}_{2} }} } \hfill &{{{\sigma }}_{{{\text{s}}_{2} }}^{2} } \hfill &{{{\sigma }}_{{{\text{s}}_{2} , {\text{s}}_{3} }} } \hfill \\ {{{\sigma }}_{{{\text{d}}_{1} , {\text{s}}_{3} }} } \hfill &{{{\sigma }}_{{{\text{d}}_{2} , {\text{s}}_{3} }} } \hfill &{{{\sigma }}_{{{\text{d}}_{3} , {\text{s}}_{3} }} } \hfill &{{{\sigma }}_{{{\text{s}}_{1} , {\text{s}}_{3} }} } \hfill &{{{\sigma }}_{{{\text{s}}_{2} , {\text{s}}_{3} }} } \hfill &{{{\sigma }}_{{{\text{s}}_{3} }}^{2} } \hfill \\ \end{array} } \right], $$

where ${{\sigma }}_{{{\text{d}}_{\text{i}} }}^{2}$ are the direct genetic variances for each trait ${\text{i}}$ = 1, 2 and 3; ${{\sigma }}_{{{\text{s}}_{\text{i}} }}^{2}$ are the indirect genetic variances for each trait ${\text{i}}$ = 1, 2 and 3; and the off-diagonal elements represent covariances, either between direct and indirect genetic effects within, ${{\sigma }}_{{{\text{d}}_{\text{i}} , {\text{s}}_{\text{i}} }} ,$ or across traits, ${{\sigma }}_{{{\text{d}}_{\text{i}} , {\text{s}}_{\text{j}} }}$; or for the same genetic effect across traits, i.e. direct genetic effects (${{\sigma }}_{{{\text{d}}_{\text{i}} ,{\text{d}}_{\text{j}} }}$) or indirect genetic effects (${{\sigma }}_{{{\text{s}}_{\text{i}} , {\text{s}}_{\text{j}} }}$).

Variance components were estimated by using Bayesian procedures. Flat priors were used for the systematic effects (${\mathbf{b}}$) and for all variance components previously described, i.e. ${\mathbf{P}}_{0}$, ${\mathbf{L}}_{0}$, ${\mathbf{G}}_{0}$ and ${\mathbf{R}}_{0}$. Marginal posterior distributions of the variance components were sampled using the Gibbs sampling algorithm, with the gibbs2f90 program [10]. Chains of 1,000,000 samples were run and the first 100,000 iterations were discarded in order to allow the algorithm to converge to the posterior distributions. Then, one sample every 10 iterations was saved. Convergence of the Markov chains was assessed by visual inspection of the trace plots. Additional file 1: Figure S1 contains the trace plots of the Markov chains for the genetic correlations and heritabilities of the three traits. Then, marginal posterior distributions of the genetic parameters (ratios of variances and correlations) and genetic responses under different evaluation procedures and economic conditions were characterized based on the saved samples of the joint posterior distribution of the variance components.

To characterize the marginal posterior distribution of response to selection (RS) stochastic simulation was used. Each replicate of the stochastic simulation was defined by three arguments: (a) the variance components used for generating the data, which was based on a saved sample from the posterior distibutions, (b) the genetic evaluation procedure, i.e. SAM or AM, and (c) the selection index used to rank candidates. The last two arguments define the relevant scenarios in our study, with the uncertainty of RS due to the uncertainty about the variance components being evaluated based on changes in the first argument across the different replicates.

Due to computational time constraints, we did not use all saved samples from the joint posterior distribution of the variance components. Instead, we used a random subset of 1000 vectors from the joint distribution to characterize the marginal posterior distributions of the expected genetic responses under different scenarios. For a particular replicate within a given scenario, the simulation to evaluate RS started by taking one sample from the posterior distribution of the variance components. Based on these variance components, new ADG, BF and FCR records were generated using a SAM that was similar to the model used for the analysis of the Duroc data, i.e. with random litter, pen, DGE and IGE effects, along with litter size, parity order, and batch effects as systematic factors. The simulations were conducted assuming an infinitesimal model [11], i.e., breeding values were sampled from a multivariate normal distribution.

For the genetic evaluation, only BF and ADG records were considered, using either the SAM or the AM. For the SAM, predictions of breeding values were obtained by solving the mixed model equations with variance components equal to those used in the simulation (best linear unbiased prediction (BLUP)). For the AM, predictions of breeding values were obtained by solving the mixed model equations associated with the AM, with variance components equal to those obtained in a previous residual maximum likelihood (REML) step (REML-BLUP) on the data generated in that replicate. The programs used to obtain the predictions of breeding values were blupf90 and remlf90 [10].

Within each scenario, the complete procedure was repeated for each one of the 1000 samples of the posterior variance components retained. The simulated datasets mimicked the management of a pig selection nucleus of 400 sows and 20 boars, where mating between close relatives, i.e. with common grand-parents, was avoided. Reproduction was organized in four batches per generation, but the genetic evaluations and selection process were performed using the first two batches of each generation. The other two batches generated information for genetic evaluation in the next generation. Each batch comprised approximately 2500 selection candidates (50% females) in pens of eight animals, litters were processed by order, thus, in general, each pen included animals from more than one litter; in general, less than 25% of the pens were formed by animals from a single litter. The best 200 females from the batch were selected and the males were selected within sire families, thus the best offspring from each sire family was selected. Given that candidates were generated in two batches the average ratio offspring/sow was 12.5

The alternative scenarios to evaluate RS were defined by the different indexes used to rank the animals. The objective was to reduce BF while increasing ADG, but with different assigned weights. When the genetic evaluation relied on REML-BLUP predictions from the AM, selection indexes were defined by assigning alternative weights to predictions of breeding values for ADG and BF (Table 2). When the SAM was used to evaluate animals (BLUP), two sets of weights were considered in the selection index, one to define the strength on one trait versus the other (ADG or BF) and the other to control the strength on DGE or IGE (Table 2). Thus, the resulting total number of scenarios assessed was the combination of these two and reached 25 (Table 2). The following equation defines the selection index value for individual $i$:

**Table 2:** Economic weights assigned in the selection index to average daily gain (ADG) and backfat thickness (BF)

	Five scenarios AM^a^	25 scenarios SAM^b^	
	$\varvec{W}_{{\varvec{ADG}}} - \varvec{W}_{{\varvec{BF}}}$ (%)	$\varvec{W}_{{\varvec{ADG}}} - \varvec{W}_{{\varvec{BF}}}$ (%)	$\varvec{W}_{{\varvec{DGE}}} - \varvec{W}_{{\varvec{IGE}}}$ (%)
Economic values	0–100	0–100	0–100
	25–75	25–75	25–75
	50–50	50–50	50–50
	75–25	75–25	75–25
	100–0	100–0	100–0

^a^The five scenarios with the classic animal model. Index for AM: $\widehat{{I_{j} }} = W_{ADG} *\widehat{{ADG_{j} }} - W_{BF} *\widehat{{BF_{j} }}$

^b^The 25 scenarios with the animal model including direct (DGE) and indirect (IGE) genetic effects are obtained by combining each element of the column $W_{DGE} - W_{IGE}$ (%)—weights on the DGE or the IGE—with the weights in the column to its left. Index for SAM: $\widehat{{I_{j} }} = W_{ADG} *\left( {W_{DGE} *\widehat{{ADG_{DGEj} }} + W_{IGE} *7 *\widehat{{ADG_{IGEj} }}} \right) - W_{BF} *\left( {W_{DGE} *\widehat{{BF_{DGEj} }} + W_{IGE} *7 *\widehat{{BF_{IGEj} }}} \right)$

2 $$ \begin{aligned} \widehat{{I_{i} }} = & W_{ADG} *\left( {W_{DGE} *\widehat{{ADG_{{DGE_{i} }} }} + W_{IGE} *7 *\widehat{{ADG_{{IGE_{i} }} }}} \right) \\ - W_{BF} *\left( {W_{DGE} *\widehat{{BF_{{DGE_{i} }} }} + W_{IGE} *7 *\widehat{{BF_{{IGE_{i} }} }}} \right), \\ \end{aligned} $$

where $\widehat{{ADG_{{DGE_{i} }} }}$, $\widehat{{ADG_{{IGE_{i} }} }}$, $\widehat{{BF_{{DGE_{i} }} }}$, and $\widehat{{BF_{{IGE_{i} }} }}$ are predicted breeding values of DGE and IGE for ADG and BF of individual $i$; $W_{DGE}$ and $W_{IGE}$ are economic weights for DGE and IGE, respectively, the number 7 represents the number of pen mates (n-1), i.e. the IGE of individual $i$ is exerted over seven pen mates; and $W_{ADG}$ and $W_{BF}$ are economic weights for ADG and BF, respectively. In order to evaluate the accuracy of the selection index, we calculated the correlations between true and predicted breeding values of the traits included in the index and the correlations between true and predicted indexes values for all scenarios.

As stated above, during data generation, ADG, BF and FCR records were obtained in the first two batches in each generation; ADG and BF information was used to take selection decisions while FCR was used just to assess the correlated response after selection for the proposed indexes. In addition, three profit traits (€), B1, B2 and B3, were derived based on three profit functions that represent three Spanish or European markets: B1 was based on a market without any constraint on BF; B2 represents a lean meat production market in which a penalty is applied if BF is outside the 6 to 10 mm range; and B3 represents a fat meat production market, in which a penalty is applied if BF is outside the 10 to 20 mm range. The general equation used to compute these profit traits was:

3 $$ B_{i} = \left( {FBW_{i} *P_{BW} } \right) - \left( {P_{pl} + FCR_{i} *\left( {FBW_{i} - IBW_{i} } \right)* P_{fd} + P_{fx} * \frac{{FBW_{i} - IBW_{i} }}{{ADG_{i} }}} \right) - PenaltyBF_{i} $$

where $B_{i}$ is the profit for individual $i$, $FBW_{i}$ is the live body weight at the end of the fattening period (assumed constant = 110 kg) of individual $i$, $P_{BW}$ is the price per kg (1.164 €/kg), $P_{pl}$ is the price of a piglet (35 €/piglet), $FCR_{i}$ is the FCR of individual $i$, $IBW_{i}$ is the initial body weight (assumed constant = 20 kg) of individual $i$, $P_{fd}$ is the price of feed (0.252 €/kg), $P_{fx}$ is the fixed cost for daily maintenance of pigs on the farm (0.09 €/d), and $PenaltyBF_{i}$ is the penalty that is applied based on BF. In both scenarios with penalties on BF (B2 and B3), the penalization is based on a price reduction per BF mm outside of the valid limits (0.012 €/mm). The assumed production costs and the prices needed to define the profit function were based on the Spanish market [12].

The simulation was run for five generations and responses for the three biological traits and the three profit traits were defined as linear regression coefficients of the average phenotype of traits across selection candidates in a given generation on generation number. One thousand regression coefficients were obtained for each trait and scenario, one for each sample from the joint posterior distribution of the variance components in the SAM. Thus, each regression coefficient can be interpreted as a sample from the marginal posterior distribution of the RS for that trait for a given scenario. Based on this procedure, we can characterize the expected RS as well as the uncertainty of this expectation, the latter being a consequence of errors in the estimates of variance components.

The simulation process was implemented in a software pipeline that combined own Fortran90 code for data generation and R code to edit the data and to compose parameter files needed to run the genetic evaluation programs (remlf90 or blupf90) and to create the list of the selected males and females, which was then read by the Fortran90 program for us as parents of the next generation. This complete pipeline package is available upon request.

The complete procedure, i.e. based on simulations using samples of the posterior distribution of the variance components from the SAM, was repeated for samples from the posterior distribution of variance components obtained from the AM. The AM had the same factors and prior structure as the SAM, except that a single additive genetic effect was fitted for each trait instead of both DGE and IGE. Thus, the genetic covariance matrix under this model was a 3 × 3 matrix. The simulated data for assessment of RS was based on an AM that fitted litter, pen, and additive genetic effects as random factors, and litter size, parity order, and batch as systematic effects. The genetic evaluation conducted for each generation was based on the same AM that generated the data by setting the variance components to those used for generating the data (BLUP). In this assessment, which did not fit social genetic effects, the same scenarios as those considered when data were generated with the SAM but the evaluation was conducted with the AM were assessed (Table 2).

## Results

For the three biological traits, we estimated genetic parameters using the SAM (see Table 3). Estimates of heritability ($h^{2}$) for ADG, BF, and FCR were moderate to high, 0.31 ± 0.09, 0.39 ± 0.10 and 0.25 ± 0.07, respectively. The ratios of total breeding value variance and total phenotypic variance ($T^{2}$) were clearly larger than $h^{2}$ for all three traits, which shows the importance of IGE for these traits. Another argument in favour of using the SAM to fit our dataset is that the deviance information criteria (DIC) value [13] associated with this model was clearly lower, than for the AM, i.e., 7268.53 versus 7524.59. For each trait, the genetic correlation between DGE and IGE (Table 3) was not statistically different from zero because the marginal posterior probabilities of the genetic correlation being higher than 0 were not greater than 0.95 or smaller than 0.05. Estimates of genetic correlations of DGE between traits (Table 3) were similar to those obtained with the AM (parameter estimates based on the AM are in Additional file 2: Table S1). The estimated genetic correlations were positive between ADG and BF and between BF and FCR, whereas the estimated correlation between ADG and FCR was not statistically different from 0. With the SAM, the only estimated genetic correlation between IGE that could be considered as statistically different from 0 was between ADG and BF (0.59 ± 0.26). For the genetic correlations between IGE and DGE, only the correlation between DGE of FCR and IGE of ADG was statistically different from 0 (0.70 ± 0.25).

**Table 3:** Posterior mean (posterior SD) of direct heritability, total heritability, genetic correlations, and variances of direct and indirect genetic effects (diagonal)

Trait	$\varvec{h}^{2}$	$\varvec{T}^{2}$	${\varvec{\upsigma}}_{{{\mathbf{Phe}}}}^{2}$	Genetic effect of traits	ADG_DGE_	ADG_IGE_	BF_DGE_	BF_IGE_	FCR_DGE_	FCR_IGE_
ADG^a^	0.31 (0.09)	0.63 (0.22)	0.75 (0.05)	ADG_DGE_	0.24 (0.08)	− 0.29 (0.25)	0.52 (0.17)*	− 0.19 (0.34)	− 0.03 (0.22)	− 0.35 (0.32)
				ADG_IGE_		4 × 10^−3^ (1 × 10^−3^)	0.24 (0.29)	0.59 (0.26)*	0.70 (0.25)*	0.18 (0.36)
BF	0.39 (0.10)	0.74 (0.27)	11.84 (0.84)	BF_DGE_			4.69 (1.24)	− 0.09 (0.36)	0.33 (0.19)*	− 0.34 (0.31)
				BF_IGE_				0.04 (0.02)	− 0.07 (0.38)	0.33 (0.38)
FCR	0.25 (0.07)	0.93 (0.43)	4.44 (0.44)	FCR_DGE_					1.10 (0.34)	− 0.17 (0.37)
				FCRI_GE_						0.03 (0.02)

Estimates obtained using the multi-trait social animal model

^a^ADG: average daily gain, BF: backfat thickness, FCR: feed conversion ratio, DGE: direct genetic effect, IGE: indirect genetic effect, $h^{2}$: ratio of direct genetic effect variance to total phenotypic variance, $T^{2}$: ratio of the total breeding value variation and the total phenotypic variance, ${{\sigma }}_{\text{Phe}}^{2}$: the total phenotypic variance

*Probability of being higher than 0 was higher than 0.95 or lower than 0.05

Table 4 shows the estimates of RS for ADG, BF, and FCR, and for B1, B2, and B3. In total, 25 scenarios were assessed based on different economic weights assigned to the traits in the selection index (ADG and BF) and to the genetic effects (DGE and IGE). In general, estimates of correlated responses in FCR were not statistically different from 0 for any scenario, i.e. the posterior probability of the response being positive or negative was less than 0.9 (positive response) or less than 0.1 (negative response). There were only two exceptions to this general pattern, which was when all the weight was allocated to the IGE ($W_{IGE}$ = 100%), with a distribution of the weight across traits of $W_{ADG}$ = 75%– $W_{BF}$ = 25% and $W_{ADG}$ = 100%– $W_{BF}$ = 0%, in these cases unfavourable positive responses (i.e. 0.07) were predicted. For responses in profit, only the profit in the lean meat production market resulted in favourable responses that were statistically different from 0, primarily for the scenarios with an economic weight of at least 50% on BF. Among these scenarios, the two best ones were when the same weight was assigned to ADG and BF and the weight on genetic effects were $W_{DGE}$ = 100%– $W_{IGE}$ = 0% and $W_{DGE}$ = 75%– $W_{IGE}$ = 25% (2.35 and 2.24 €/pig, respectively). Unfavourable economic responses were estimated when all the weight was assigned to the IGE ($W_{DGE}$ = 0%– $W_{IGE}$ = 100%) and the distributions of this weight across traits were $W_{ADG}$ = 75%– $W_{BF}$ = 25% and $W_{ADG}$ = 100%– $W_{BF}$ = 0% (− 3.26 and -− 3.34 €/pig respectively). In these two scenarios, unfavourable responses on FCR were also estimated. Table 4 includes the accuracy of the index predictions, i.e. the correlations between predicted and true values for the indexes. As the weight was moved from IGE to DGE, the accuracy of the indexes increased from 0.54–0.58 to 0.63–0.70, depending on the weights assigned to the traits. In general, accuracy of the indexes also increased when the weight assigned to BF increased. More details are in Additional file 3: Tables S2, S3 and S4, which also present the accuracies of predictions of IGE, DGE and TBV.

**Table 4:** Posterior mean (posterior SD) of the responses to selection for 25 indexes

$\varvec{W}_{{\varvec{ADG}}} - \varvec{W}_{{\varvec{BF}}}$, %^a^	0–100	25–75	50–50	75–25	100–0
$\varvec{W}_{{\varvec{DGE}}} - \varvec{W}_{{\varvec{IGE}}}$, %	0–100	0–100	0–100	0–100	0–100
ADG, kg	− 0.01 (0.02)	0.00 (0.02)	0.01 (0.02)	0.02 (0.02)	0.02 (0.02)
BF, mm	− 0.88 (1.24)	− 0.56 (1.26)	0.74 (0.97)	1.50 (0.97)*	1.54 (0.99)*
FCR, kg/kg	− 0.04 (0.05)	− 0.02 (0.06)	0.03 (0.05)	0.07 (0.05)*	0.07 (0.05)*
B1, €	0.71 (1.26)	0.44 (1.32)	− 0.62 (1.26)	− 1.28 (1.16)	− 1.31 (1.13)
B2, €	1.53 (1.82)	0.93 (1.93)	− 1.62 (1.42)	− 3.26 (1.67)*	− 3.34 (1.71)*
B3, €	0.46 (1.37)	0.17 (1.43)	− 1.30 (1.22)	− 2.64 (1.52)*	− 2.72 (1.54)*
$\rho (I,\widehat{I})$	0.54 (0.08)	0.54 (0.08)	0.54 (0.09)	0.58 (0.08)	0.57 (0.08)

$\varvec{W}_{{\varvec{ADG}}} - \varvec{W}_{{\varvec{BF}}}$, %	0–100	25–75	50–50	75–25	100–0
$\varvec{W}_{{\varvec{DGE}}} - \varvec{W}_{{\varvec{IGE}}}$, %	25–75	25–75	25–75	25–75	25–75
ADG, kg	− 0.03 (0.02)*	− 0.02 (0.02)	0.01 (0.02)	0.04 (0.02)*	0.04 (0.02)*
BF, mm	− 2.02 (0.99)*	− 1.83 (1.03)*	0.02 (0.75)	1.84 (0.88)*	2.01 (0.89)*
FCR, kg/kg	− 0.03 (0.05)	− 0.02 (0.05)	0.03 (0.05)	0.05 (0.05)	0.05 (0.05)
B1, €	0.20 (1.25)	0.09 (1.26)	− 0.54 (1.20)	− 0.81 (1.16)	− 0.75 (1.16)
B2, €	2.01 (1.44)*	1.78 (1.50)	− 0.59 (1.28)	− 3.23 (1.65)*	− 3.40 (1.70)*
B3, €	− 0.31 (1.41)	− 0.33 (1.41)	− 0.69 (1.17)	− 2.53 (1.55)*	− 2.68 (1.61)*
$\rho (I,\widehat{I})$	0.57 (0.08)	0.56 (0.08)	0.49 (0.09)	0.56 (0.07)	0.57 (0.07)

$\varvec{W}_{{\varvec{ADG}}} - \varvec{W}_{{\varvec{BF}}}$, %	0–100	25–75	50–50	75–25	100–0
$\varvec{W}_{{\varvec{DGE}}} - \varvec{W}_{{\varvec{IGE}}}$, %	50–50	50–50	50–50	50–50	50–50
ADG, kg	− 0.04 (0.02)*	− 0.03 (0.02)*	0.01 (0.01)*	0.05 (0.01)*	0.05 (0.01)*
BF, mm	− 2.45 (0.74)*	− 2.41 (0.73)*	− 0.87 (0.45)*	1.54 (0.86)*	1.93 (0.81)*
FCR, kg/kg	− 0.02 (0.05)	− 0.02 (0.05)	− 0.01 (0.05)	0.01 (0.05)	0.01 (0.05)
B1, €	− 0.27 (1.25)	− 0.11 (1.26)	0.48 (1.13)	0.35 (1.1)	0.28 (1.16)
B2, €	1.81 (1.29)*	1.97 (1.31)*	1.55 (1.28)	− 1.68 (1.71)	− 2.27 (1.73)*
B3, €	− 0.99 (1.37)	− 0.79 (1.39)	0.56 (1.12)	− 1.02 (1.57)	− 1.53 (1.66)
$\rho (I,\widehat{I})$	0.65 (0.07)	0.65 (0.07)	0.58 (0.07)	0.60 (0.07)	0.61 (0.06)

$\varvec{W}_{{\varvec{ADG}}} - \varvec{W}_{{\varvec{BF}}}$, %	0–100	25–75	50–50	75–25	100–0
$\varvec{W}_{{\varvec{DGE}}} - \varvec{W}_{{\varvec{IGE}}}$, %	75–25	75–25	75–25	75–25	75–25
ADG, kg	− 0.04 (0.02)*	− 0.03 (0.02)*	0.00 (0.01)	0.04 (0.02)*	0.04 (0.02)*
BF, mm	− 2.35 (0.76)*	− 2.32 (0.77)*	− 1.21 (0.65)*	0.74 (0.95)	1.29 (0.89)*
FCR, kg/kg	− 0.01 (0.06)	− 0.02 (0.06)	− 0.04 (0.06)	− 0.03 (0.05)	− 0.02 (0.05)
B1, €	− 0.49 (1.27)	− 0.16 (1.32)	0.85 (1.23)	1.09 (1.07)	0.98 (1.06)
B2, €	1.56 (1.30)*	1.88 (1.35)*	2.24 (1.48)*	0.11 (1.77)	− 0.72 (1.70)
B3, €	− 1.14 (1.41)	− 0.79 (1.43)	0.83 (1.21)	0.44 (1.42)	− 0.13 (1.51)
$\rho (I,\widehat{I})$	0.69 (0.06)	0.70 (0.06)	0.65 (0.07)	0.63 (0.07)	0.63 (0.06)

$\varvec{W}_{{\varvec{ADG}}} - \varvec{W}_{{\varvec{BF}}}$, %	0–100	25–75	50–50	75–25	100–0
$\varvec{W}_{{\varvec{DGE}}} - \varvec{W}_{{\varvec{IGE}}}$, %	100–0	100–0	100–0	100–0	100–0
ADG, kg	− 0.04 (0.02)*	− 0.03 (0.02)*	0.00 (0.01)	0.03 (0.02)*	0.04 (0.02)*
BF, mm	− 2.24 (0.80)*	− 2.23 (0.81)*	− 1.38 (0.80)*	0.26 (1.03)	0.84 (0.96)
FCR, kg/kg	0.00 (0.06)	− 0.01 (0.06)	− 0.04 (0.06)	− 0.05 (0.05)	− 0.04 (0.05)
B1, €	− 0.61 (1.28)	− 0.22 (1.34)	0.86 (1.26)	1.33 (1.12)	1.27 (1.05)
B2, €	1.40 (1.31)	1.78 (1.37)*	2.35 (1.52)*	0.95 (1.83)	0.15 (1.77)
B3, €	− 1.18 (1.42)	− 0.79 (1.46)	0.74 (1.24)	0.94 (1.34)	0.53 (1.42)
$\rho (I,\widehat{I})$	0.69 (0.06)	0.70 (0.06)	0.66 (0.07)	0.63 (0.07)	0.63 (0.06)

Data were simulated using variance component samples from the marginal posterior distribution of the social animal model and the responses were obtained in five generations of selection evaluating candidates using the social animal model

^a^ADG: average daily gain, BF: backfat thickness, FCR: feed conversion ratio, $W_{ADG} - W_{BF}$: proportion of economic weight assigned to ADG and BF in the selection index, $W_{DGE} - W_{IGE}$: proportion of economic weight assigned to direct (DGE) and indirect (IGE) genetic effects of traits in the selection index, B1: economic benefit in a non-BF-constrained market, B2: economic benefit with BF penalty out of the range 6 to 10 mm, B3: economic benefit with BF penalty out of the range 10 to 20 mm, $\rho (I,\widehat{I})$: correlation between predicted $\left( {\widehat{I}} \right)$ and true value ($I$) of the index

*Probability of being higher than 0 was higher than 0.95 or lower than 0.05

When the data were generated using the SAM and genetic evaluations were based on the AM, the overall pattern of correlated responses in FCR was similar to that described for genetic evaluations based on the SAM (Table 5). Estimated responses in FCR were not statistically different from 0 for any distribution of economic weight between BF and ADG. For profit traits, only B2 had responses that were statistically different from 0, which occurred when the weight on BF was at least 50%, i.e. 2.02, 1.72 and 1.43 €/pig, for weights on BF of 50, 75 and 100%, respectively. The pattern of the correlations between the predicted values of the indexes and the real values of the index computed based on TBV predictions (Table 5) was also similar to that of the accuracies of the index when data were both simulated and analysed with the SAM (Table 4). These correlations increased as the weight assigned to BF increased and were comparable to those reported in Table 4 when the same weight was assigned to IGE and DGE ($W_{IGE}$ = 50 and $W_{DGE}$ = 50). As expected, these correlations were higher when the SAM (Table 4) was used for the genetic evaluation than when the AM was used (Table 5). These higher correlations for SAM have consequences on direct responses for ADG and BF, which were slightly higher when the SAM was used for genetic evaluation (Table 4). However, it should be noted that the data were generated using the SAM for both these cases.

**Table 5:** Posterior mean (posterior SD) of the responses to selection for five indexes

*W*_*ADG*_–*W*_*BF*_, %^a^	0–100	25–75	50–50	75–25	100–0
ADG, kg	− 0.04 (0.02)*	− 0.03 (0.02)*	0.00 (0.01)	0.04 (0.02)*	0.04 (0.02)*
BF, mm	− 2.29 (0.74)*	− 2.29 (0.74)*	− 1.21 (0.66)*	0.86 (0.94)	1.36 (0.85)*
FCR, kg/kg	0.00 (0.05)	− 0.01 (0.05)	− 0.03 (0.05)	− 0.02 (0.05)	− 0.02 (0.04)
B1, €	− 0.62 (1.20)	− 0.34 (1.25)	0.64 (1.17)	0.96 (1.01)	0.89 (0.99)
B2, €	1.43 (1.22)*	1.72 (1.28)*	2.02 (1.41)*	− 0.18 (1.67)	− 0.90 (1.59)
B3, €	− 1.21 (1.35)	− 0.92 (1.38)	0.62 (1.14)	0.22 (1.34)	− 0.28 (1.42)
$\rho (I_{TBV} ,\widehat{I})$	0.60 (0.09)	0.60 (0.09)	0.53 (0.1)	0.52 (0.1)	0.54 (0.1)

Data were simulated using variance component samples from the marginal posterior distribution of the social animal model and the responses were obtained in five generations of selection evaluating candidates using the classical animal model

^b^ADG: average daily gain, BF: backfat thickness, FCR: feed conversion ratio, $W_{ADG} - W_{BF}$: proportion of economic weight assigned to ADG and BF in the selection index, B1: economic benefit in a non-BF-constrained market, B2: economic benefit with BF penalty out of the range 6 to 10 mm, B3: economic benefit with BF penalty out of the range 10 to 20 mm, $\rho (I,\widehat{I})$: correlation between predicted $\left( {\widehat{I}} \right)$ index value and true value of the total breeding value (TBV)

*Probability of being higher than 0 was higher than 0.95 or lower than 0.05

As a baseline situation, we explored the genetic responses when IGE were ignored during the process of data generation (i.e. AM) and also during the genetic evaluation of the candidates, which was conducted by applying an AM with the variance components set to values that were used for the simulation in each replicate (Table 6). The most remarkable feature of this baseline situation was the decrease in the magnitude of the standard deviations of the marginal posterior distributions of the responses compared to those in Tables 4 and 5. In this case, the maximum value of the ratio between posterior standard deviation and posterior mean, across traits and scenarios, was 29.7, while it was only 9.3 when the SAM was used for simulation and genetic evaluations was conducted using the AM (Table 5) and as high as 37.5 when the genetic evaluation was done with the SAM (Table 4). When data were simulated using the AM, responses in FCR were statistically different from 0 for the two scenarios in which most of the weight was assigned to BF. In these two scenarios, favourable economic responses were also estimated not only for markets with and without constraints on BF (3.04 and 3.05 €/pig for B2 and 0.92 €/pig and 0.96 €/pig for B1). In addition, positive economic responses were obtained in the scenario in which ADG and BF had similar weights for both B1 and B2 (0.70 €/pig and 2.09 €/pig, respectively), but in this case the response in FCR was not statistically different from 0. In the baseline scenario, the accuracy of the selection indexes ranged from 0.58 to 0.69, and the largest accuracies were obtained in the scenarios where most of the weight was assigned to BF.

**Table 6:** Posterior mean (posterior SD) of the responses to selection for five indexes when data were generated with the animal model

*W*_*ADG*_–*W*_*BF*_, %^a^	0–100	25–75	50–50	75–25	100–0
ADG, kg	− 0.01 (0.01)	− 0.01 (0.01)	0.01 (0.01)*	0.03 (0.01)*	0.03 (0.01)*
BF, mm	− 2.11 (0.56)*	− 2.04 (0.55)*	− 1.15 (0.52)*	0.44 (0.94)	0.95 (0.87)
FCR, kg/kg	− 0.05 (0.02)*	− 0.05 (0.02)*	− 0.02 (0.03)	0.01 (0.04)	0.02 (0.04)
B1, €	0.92 (0.60)*	0.96 (0.61)*	0.70 (0.74)*	0.03 (0.89)	− 0.22 (0.85)
B2, €	3.04 (0.75)*	3.05 (0.77)*	2.09 (1.11)*	− 0.56 (1.89)	− 1.48 (1.79)
B3, €	0.53 (0.57)	0.61 (0.60)	0.74 (0.71)*	− 0.42 (1.34)	− 1.03 (1.42)
$\rho (I,\widehat{I})$	0.67 (0.07)	0.69 (0.07)	0.61 (0.11)	0.58 (0.08)	0.58 (0.07)

Data were simulated using variance component samples from the marginal posterior distribution of the classical animal model and the responses were obtained in five generations of selection evaluating candidates using also the classical animal model

^a^ADG: average daily gain, BF: backfat thickness, FCR: feed conversion ratio, $W_{ADG} - W_{BF}$: proportion of economic weight assigned to ADG and BF in the selection index, B1: economic benefit in a non-BF-constrained market, B2: economic benefit with BF penalty out of the range 6 to 10 mm, B3: economic benefit with BF penalty out of the range 10 to 20 mm, $\rho (I,\widehat{I})$: correlation between predicted $\left( {\widehat{I}} \right)$ and true value ($I$) of the index

*Probability of being higher than 0 was higher than 0.95 or lower than 0.05

## Discussion

In general, genetic parameters estimated with the SAM are subject to large estimation errors. In our case, these errors were particularly large because of the small size of our dataset, but other studies that used much larger datasets reported a similar magnitude of errors [7, 14–17]. Thus, this could be due to the limited amount of information in pig datasets to separate direct from indirect genetic effects, as well as the within-pen sum of indirect genetic effects from other factors such as pen effects [18]. Consequently, the assessment of response to selection when a SAM is proposed to evaluate selection candidates has to take such estimation errors into account. To accomplish this, we used Monte Carlo methods to integrate variation of the posterior distribution of the variance components on genetic response predictions out. Since the function to assess the genetic response is complex, i.e. based on stochastic simulation, we took advantage of the availability of a computational cluster to afford the computations.

As expected, the uncertainty of the expected responses was much larger when the SAM was used for generating the data and for evaluation than when the AM was used. Direct responses in ADG and BF were only slightly smaller when the AM was used to rank the candidates (Table 5) than when the SAM was used to rank the candidates and the same weight was assigned to both IGE and DGE (Table 4). These findings indicate that using a different model to that used for data generation is not the most important factor for a decrease in accuracies and responses. The fact that similar patterns of direct responses in ADG and BF were observed when the evaluation was conducted using the SAM or AM could be explained by the large uncertainty on the model parameters for generating the data, which dominated the results for both models. In situations where these errors would have a lower magnitude, using the wrong model for genetic evaluation is expected to have a stronger impact. Previous studies have addressed the theoretical magnitude of the estimation errors of genetic parameters with the SAM and reported difficulties associated with estimations based on such models [14, 18, 19]. However, to the best of our knowledge, none of these studies have addressed the consequences that these large estimation errors can have on the expected genetic responses to selection.

Our initial hypothesis was that using the SAM for the genetic evaluation would allow us to take advantage of genetic correlations among DGE and IGE of traits, which might help to alleviate the unfavorable genetic correlations that exist between BF and ADG, or between ADG and FCR: for example, a negative genetic correlation between IGE of FCR and DGE of ADG. Based on our results, this hypothesis does not hold for the population under study because the highest economic responses were obtained when the AM was used for genetic evaluation (Table 6). As mentioned, this result can be explained by the fact that the use of a complex model introduces a large uncertainty on genetic parameter estimates, which translates to uncertainty in responses to selection. This, in turn, prevented responses from the SAM, which were of similar magnitude to those estimated when the AM was used, to be declared as significantly different from 0. This larger uncertainty on model parameters of the more complex models also reduces prediction accuracies (Tables 4, 5 and 6), and see Additional file 3: Table S2, particularly when most of the weight is assigned to IGE or to ADG, which also has consequences on observed responses to selection.

In spite of the overall rejection of our hypothesis, it should be noted that when the genetic evaluation was conducted using the SAM (Table 4), the best economic responses were achieved when both BF and ADG had the same weight but the selection index relied exclusively on DGE. Note that, in the simulation, phenotypes were generated considering the concept of total breeding value (DGE + (n-1)*IGE). This result partially supports our hypothesis, in the sense that the largest correlated response on FCR was obtained when the weights on DGE and IGE were different from their weights on TBV, i.e. 0 versus (n-1) on IGE.

Another point that could explain the failure of our initial hypothesis is that the correlations that could eliminate the antagonistic relationship between traits did not reach a relevant magnitude in the population under study. This could happen, for example, if the correlation of IGE for ADG with DGE for BF was low. Thus, one trait could be modified by selecting on the IGE and the other by selecting on DGE. Then, a correlated response in FCR could be expected if the correlation between IGE for ADG and IGE for FCR was negative and that between DGE of BF and DGE of FCR was positive. Some of these requirements necessary for our hypothesis to hold were fulfilled in our case, i.e., the posterior mean of the correlation between IGE for ADG and DGE for BF was lower than that of the correlation between DGE for the two traits. However, other requirements were not satisfied. The correlations mentioned just above are only a part of the whole correlation structure, for example those between IGE and DGE across traits should be also considered. Accounting for the whole structure is the reason why we conducted an assessment of responses based on simulation to explore the consequences of different selection indexes combining IGE and DGE.

Regarding the applied problem addressed in our study, i.e. indirect improvement of FCR by joint selection on ADG and BF, our results indicate that, in this Duroc pig population, response in FCR is highly driven by a reduction in BF, and selecting only on ADG by ignoring BF, would lead to economic losses. When the data were generated with the AM, the economic responses resulted from both a direct reduction in BF and a correlated response in FCR. However, when the SAM was used to generate the data, the achieved economic benefit did not result from improved FCR but only from a reduction in BF, which was of value in some of the markets assessed.

The magnitude of genetic parameters is known to depend on the population under study, and for the traits considered here, a wide range of estimates have been reported in the literature for different lines and breeds. Although the genetic correlation between ADG and BF tends to be positive [4, 20], some studies on other Duroc lines have reported correlation values of nearly 0 [21]. Estimates of genetic correlations between BF and FCR and between ADG and FCR show a wider range of variability, with both having both negative and positive estimates [4, 20, 21]. In [4, 21], the observed responses in the implemented selection experiments clearly matched the estimated parameters. Thus, we can state that the general pattern of the genetic correlations that we estimated, both for IGE and DGE, is compatible with previously reported estimates of genetic parameters, although they were obtained with the AM.

## Conclusions

Using the SAM in genetic evaluation to indirectly improve FCR by selection on ADG and BF does not overcome the unfavourable genetic correlations that exist between the traits when they are evaluated with the traditional AM. On the one hand, this is due to the large magnitude of the estimation errors of the genetic parameters estimated in more complex models such as the SAM. On the other hand, the correlations between IGE and DGE that could help overcome the unfavourable genetic correlations between DGE did not reach sufficiently high magnitudes.

## **Supplementary information**

**Additional file 1: Figure S1.** Trace plots of Markov chains of the genetic parameters for the social animal model. This file contains the trace plots of the Markov chains for the genetic correlations and heritabilities of ADG, BF and FCR. This can be used to assess that our Markov chains have an acceptable rate of mixing.

**Additional file 2: Table S1.** Posterior mean (posterior SD) of genetic (above diagonal) and phenotypic (below diagonal) correlations, and heritabilities (diagonal). This table contains the estimated genetic parameters for ADG, BF and FCR using the AM. These estimates can be compared to those obtained when the SAM (Table 3) was used for the analysis of the available dataset.

**Additional file 3: Table S2.** Posterior mean (posterior SD) of the correlation between true and predicted breeding values using the SAM. This table contains the correlations between true and predicted values of DGE and IGE of traits included in the selection index (ADG and BF) for the 25 studied scenarios using the SAM. This table also contains the correlations between true and predicted values of an index calculated based on the total breeding value definition of the involved traits. **Table S3.** Posterior mean (posterior SD) of the correlations between true figures for total breeding value, direct and indirect genetic effects, and breeding values predictions obtained with the AM. This table contains the correlations between predicted values of EBV using the AM and the true values of DGE, IGE, and TBV used in the simulation, using the SAM, for the traits included in the selection index (ADG and BF). **Table S4.** Posterior mean (posterior SD) of the correlations between true and predicted breeding values using the AM for data generation and evaluation. This table contains the correlations between true and predicted values of EBV for traits included in the selection index (ADG and BF) when both simulation and genetic evaluations are conducted using the AM.
