# Supplementary material for: Selection for feed efficiency using the social effects animal model in growing Duroc pigs: evaluation by simulation
Source: Genet Sel Evol. 2020 Sep 29;52:53. doi: 10.1186/s12711-020-00572-4 (PMC7526410; doi:10.1186/s12711-020-00572-4)
Supplement: Supplementary file 1 — Additional file 1: Figure S1. Trace plots of Markov chains of the genetic parameters for the social animal model. This file contains the trace plots of the Markov chains for the genetic correlations and heritabilities of ADG, BF and FCR. This can be used to assess that our Markov chains have an acceptable rate of mixing. [file 12711_2020_572_MOESM1_ESM.pdf]

Additional file 1

Figure S1 Trace plots of Markov Chains of the genetic parameters for the social animal model

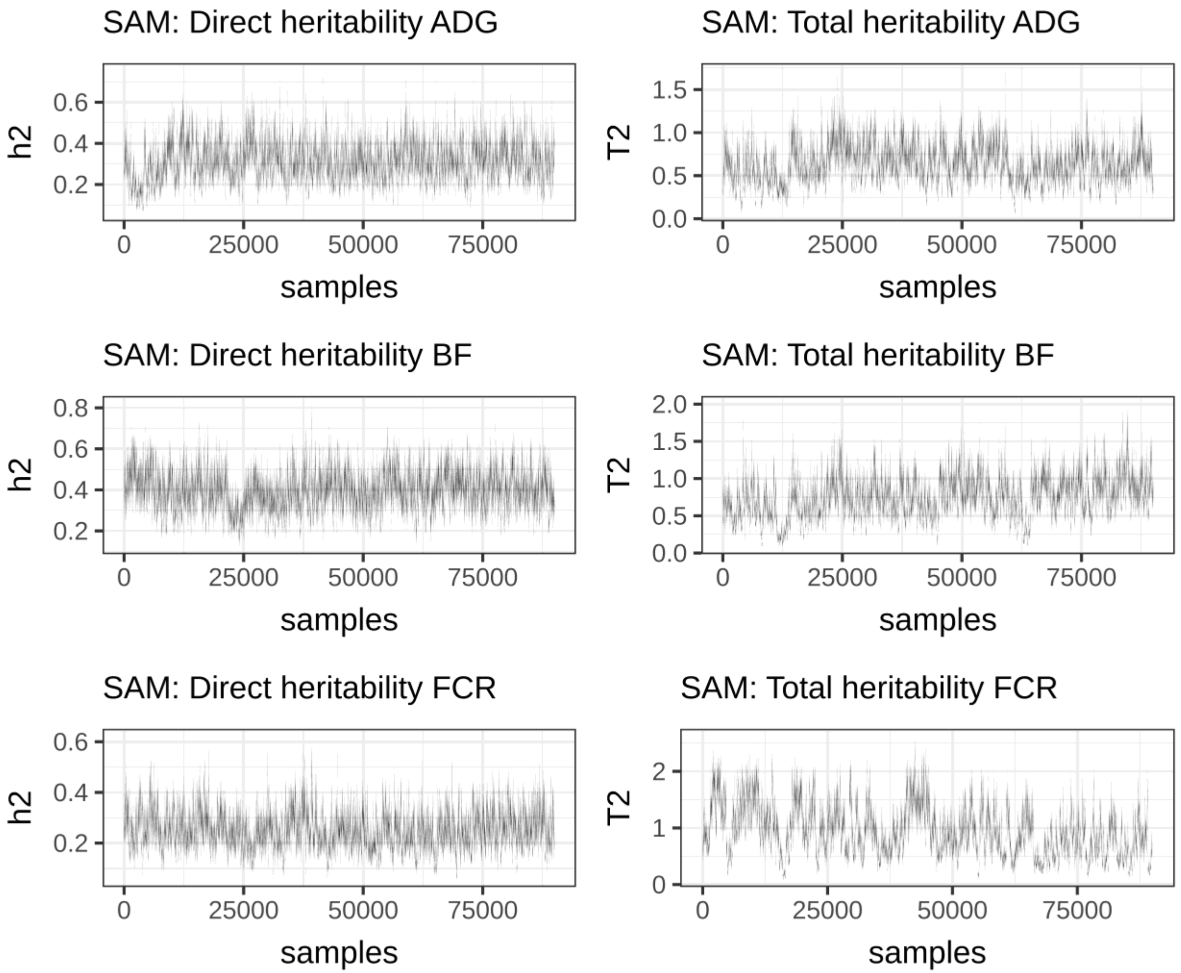

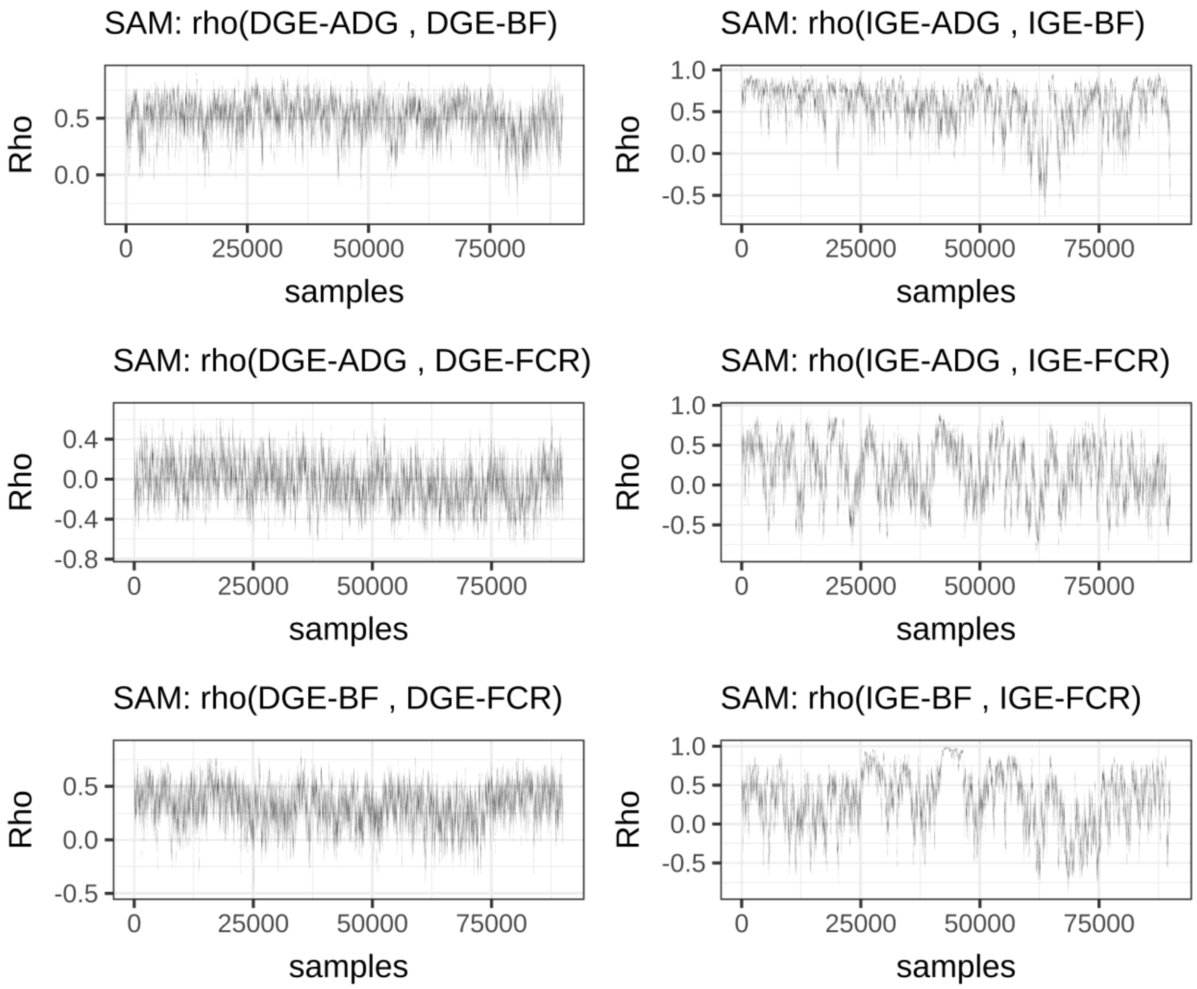
SAM: rho(DGE-ADG , DGE-BF)
Rho
0.5
0.0
0
25000
50000
75000
samples
SAM: rho(IGE-ADG , IGE-BF)
Rho
1.0
0.5
0.0
-0.5
0
25000
50000
75000
samples
SAM: rho(DGE-ADG , DGE-FCR)
Rho
0.4
0.0
-0.4
-0.8
0
25000
50000
75000
samples
SAM: rho(IGE-ADG , IGE-FCR)
Rho
1.0
0.5
0.0
-0.5
0
25000
50000
75000
samples
SAM: rho(DGE-BF , DGE-FCR)
Rho
0.5
0.0
-0.5
0
25000
50000
75000
samples
SAM: rho(IGE-BF , IGE-FCR)
Rho
1.0
0.5
0.0
-0.5
0
25000
50000
75000
samples

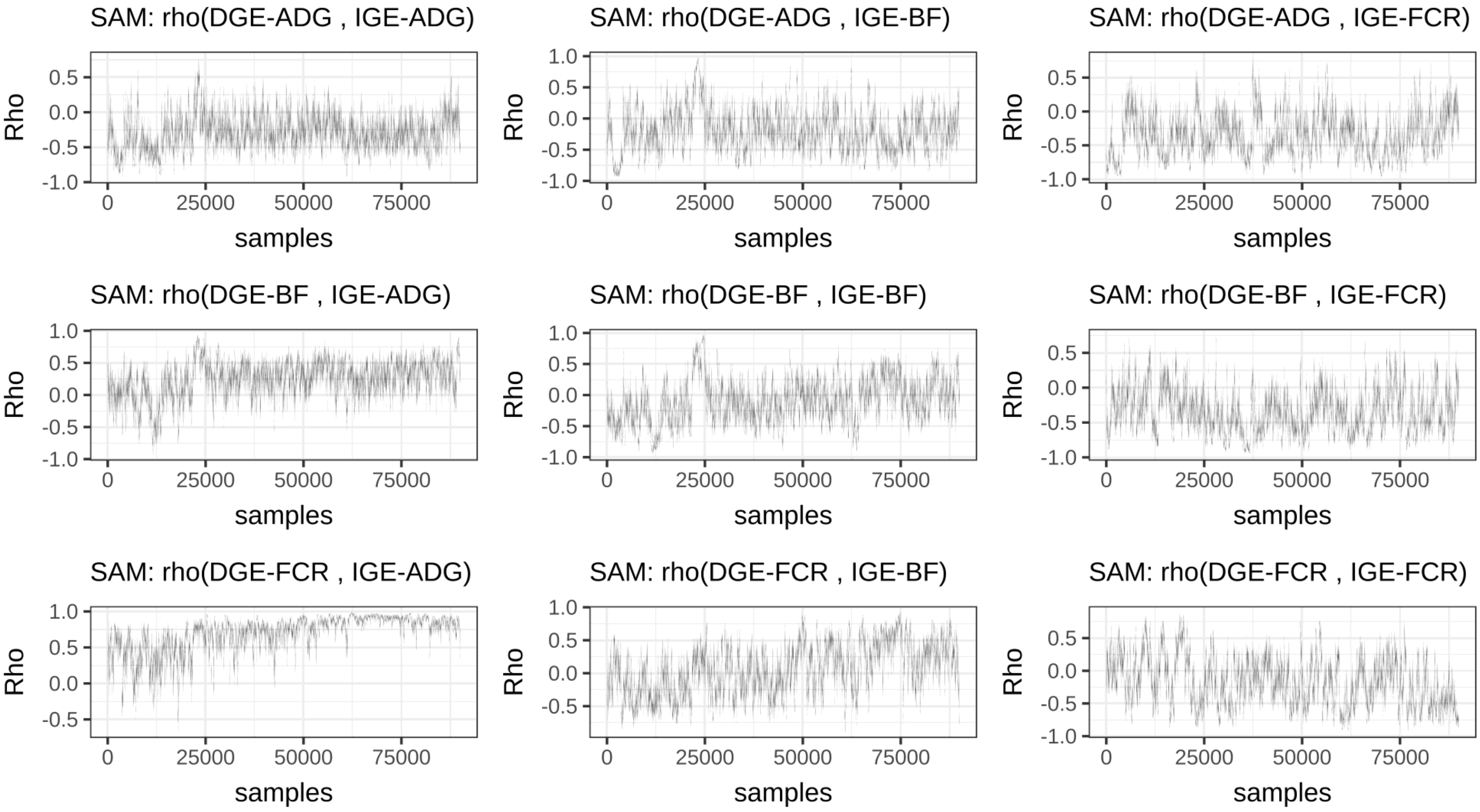

SAM: rho(DGE-ADG , IGE-ADG)
SAM: rho(DGE-ADG , IGE-BF)
SAM: rho(DGE-ADG , IGE-FCR)
SAM: rho(DGE-BF , IGE-ADG)
SAM: rho(DGE-BF , IGE-BF)
SAM: rho(DGE-BF , IGE-FCR)
SAM: rho(DGE-FCR , IGE-ADG)
SAM: rho(DGE-FCR , IGE-BF)
SAM: rho(DGE-FCR , IGE-FCR)
Rho
samples
0
25000
50000
75000
1.0
0.5
0.0
-0.5
-1.0
