# Supplementary material for: Selection for feed efficiency using the social effects animal model in growing Duroc pigs: evaluation by simulation
Source: Genet Sel Evol. 2020 Sep 29;52:53. doi: 10.1186/s12711-020-00572-4 (PMC7526410; doi:10.1186/s12711-020-00572-4)
Supplement: Supplementary file 2 — Additional file 2: Table S1. Posterior mean (posterior SD) of genetic (above diagonal) and phenotypic (below diagonal) correlations, and heritabilities (diagonal). This table contains the estimated genetic parameters for ADG, BF and FCR using the AM. These estimates can be compared to those obtained when the SAM (Table 3) was used for the analysis of the available dataset. [file 12711_2020_572_MOESM2_ESM.docx]

**Additional file 2:**

**Table S1** Posterior Mean (Posterior SD)^a^ of genetic (above diagonal) and phenotypic (below diagonal) correlations, and heritabilities (diagonal)

|  | ADG ^b^ | BF | FCR |
| --- | --- | --- | --- |
| ADG | 0.20(0.08) | 0.50(0.24)* | 0.46(0.46) |
| BF | 0.59(0.03)* | 0.37(0.10) | 0.65(0.27)* |
| FCR | -0.04(0.04) | 0.31(0.03)* | 0.18(0.10) |

^a^Estimates obtained using the classical multi-trait animal model

^b^ADG: average daily gain, BF: backfat thickness, FCR: feed conversion ratio.

* Probability of being higher than 0 was not higher than 0.95 or lower than 0.05.
