# Supplementary material for: Selection for feed efficiency using the social effects animal model in growing Duroc pigs: evaluation by simulation
Source: Genet Sel Evol. 2020 Sep 29;52:53. doi: 10.1186/s12711-020-00572-4 (PMC7526410; doi:10.1186/s12711-020-00572-4)
Supplement: Supplementary file 3 — Additional file 3: Table S2. Posterior mean (posterior SD) of the correlation between true and predicted breeding values using the SAM. This table contains the correlations between true and predicted values of DGE and IGE of traits included in the selection index (ADG and BF) for the 25 studied scenarios using the SAM. This table also contains the correlations between true and predicted values of an index calculated based on the total breeding value definition of the involved traits. Table S3. Posterior mean (posterior SD) of the correlations between true figures for total breeding value, direct and indirect genetic effects, and breeding values predictions obtained with the AM. This table contains the correlations between predicted values of EBV using the AM and the true values of DGE, IGE, and TBV used in the simulation, using the SAM, for the traits included in the selection index (ADG and BF). Table S4. Posterior mean (posterior SD) of the correlations between true and predicted breeding values using the AM for data generation and evaluation. This table contains the correlations between true and predicted values of EBV for traits included in the selection index (ADG and BF) when both simulation and genetic evaluations are conducted using the AM. [file 12711_2020_572_MOESM3_ESM.docx]

**Additional file 3**

**Table S2** Posterior mean (posterior SD) of the correlation between true and predicted breeding values using SAM^a^

| W_ADG_ – W_BF_, % ^b^ | 0 – 100 | 25 – 75 | 50 – 50 | 75 – 25 | 100 – 0 |
| --- | --- | --- | --- | --- | --- |
| W_DGE_ – W_IGE_, % | 0 – 100 | 0 – 100 | 0 – 100 | 0 – 100 | 0 – 100 |
| $\rho({DGE}_{ADG},\hat{{DGE}_{ADG})}$ | 0.69(0.06) | 0.68(0.06) | 0.69(0.06) | 0.69(0.06) | 0.69(0.06) |
| ${\rho(IGE}_{ADG},\hat{{IGE}_{ADG})}$ | 0.61(0.07) | 0.62(0.08) | 0.62(0.08) | 0.58(0.07) | 0.57(0.08) |
| ${\rho(TBV}_{ADG},\hat{{TBV}_{ADG})}$ | 0.61(0.07) | 0.62(0.08) | 0.62(0.08) | 0.58(0.07) | 0.57(0.08) |
| ${\rho(DGE}_{BF},\hat{{DGE}_{BF})}$ | 0.74(0.06) | 0.74(0.06) | 0.73(0.06) | 0.74(0.05) | 0.74(0.06) |
| ${\rho(IGE}_{BF},\hat{{IGE}_{BF})}$ | 0.54(0.08) | 0.54(0.08) | 0.59(0.08) | 0.59(0.07) | 0.57(0.07) |
| ${\rho(TBV}_{BF},\hat{{TBV}_{BF})}$ | 0.54(0.08) | 0.54(0.08) | 0.59(0.08) | 0.59(0.07) | 0.57(0.07) |
| $\rho(I_{TBV},\hat{I)}$ | 0.25(0.24) | 0.20(0.27) | 0.00(0.25) | 0.18(0.19) | 0.25(0.18) |
| W_ADG_ – W_BF_, % | 0 – 100 | 25 – 75 | 50 – 50 | 75 – 25 | 100 – 0 |
| W_DGE_ – W_IGE_, % | 25 – 75 | 25 – 75 | 25 – 75 | 25 – 75 | 25 – 75 |
| $\rho({DGE}_{ADG},\hat{{DGE}_{ADG})}$ | 0.69(0.06) | 0.69(0.06) | 0.69(0.06) | 0.69(0.06) | 0.69(0.06) |
| ${\rho(IGE}_{ADG},\hat{{IGE}_{ADG})}$ | 0.60(0.07) | 0.61(0.08) | 0.63(0.08) | 0.59(0.07) | 0.59(0.07) |
| ${\rho(TBV}_{ADG},\hat{{TBV}_{ADG})}$ | 0.60(0.07) | 0.61(0.07) | 0.63(0.08) | 0.58(0.07) | 0.57(0.07) |
| ${\rho(DGE}_{BF},\hat{{DGE}_{BF})}$ | 0.73(0.06) | 0.73(0.06) | 0.74(0.06) | 0.73(0.06) | 0.72(0.06) |
| ${\rho(IGE}_{BF},\hat{{IGE}_{BF})}$ | 0.56(0.07) | 0.55(0.08) | 0.59(0.08) | 0.58(0.08) | 0.58(0.07) |
| ${\rho(TBV}_{BF},\hat{{TBV}_{BF})}$ | 0.57(0.08) | 0.57(0.08) | 0.63(0.08) | 0.60(0.08) | 0.59(0.08) |
| $\rho(I_{TBV},\hat{I)}$ | 0.51(0.16) | 0.49(0.17) | 0.26(0.21) | 0.40(0.14) | 0.46(0.13) |
| W_ADG_ – W_BF_, % | 0 – 100 | 25 – 75 | 50 – 50 | 75 – 25 | 100 – 0 |
| W_DGE_ – W_IGE_, % | 50 – 50 | 50 – 50 | 50 – 50 | 50 – 50 | 50 – 50 |
| $\rho({DGE}_{ADG},\hat{{DGE}_{ADG})}$ | 0.69(0.06) | 0.69(0.06) | 0.68(0.07) | 0.66(0.06) | 0.67(0.06) |
| ${\rho(IGE}_{ADG},\hat{{IGE}_{ADG})}$ | 0.61(0.07) | 0.61(0.07) | 0.63(0.07) | 0.63(0.07) | 0.63(0.07) |
| ${\rho(TBV}_{ADG},\hat{{TBV}_{ADG})}$ | 0.64(0.06) | 0.66(0.06) | 0.67(0.07) | 0.62(0.06) | 0.61(0.06) |
| ${\rho(DGE}_{BF},\hat{{DGE}_{BF})}$ | 0.70(0.06) | 0.71(0.06) | 0.75(0.06) | 0.73(0.06) | 0.72(0.06) |
| ${\rho(IGE}_{BF},\hat{{IGE}_{BF})}$ | 0.59(0.07) | 0.58(0.07) | 0.59(0.08) | 0.59(0.08) | 0.59(0.08) |
| ${\rho(TBV}_{BF},\hat{{TBV}_{BF})}$ | 0.65(0.07) | 0.66(0.07) | 0.71(0.07) | 0.69(0.07) | 0.68(0.07) |
| $\rho(I_{TBV},\hat{I)}$ | 0.65(0.07) | 0.65(0.07) | 0.58(0.07) | 0.60(0.07) | 0.61(0.06) |
| W_ADG_ – W_BF_, % | 0 – 100 | 25 – 75 | 50 – 50 | 75 – 25 | 100 – 0 |
| W_DGE_ – W_IGE_, % | 75 – 25 | 75 – 25 | 75 – 25 | 75 – 25 | 75 – 25 |
| $\rho({DGE}_{ADG},\hat{{DGE}_{ADG})}$ | 0.69(0.06) | 0.70(0.06) | 0.68(0.06) | 0.64(0.06) | 0.64(0.06) |
| ${\rho(IGE}_{ADG},\hat{{IGE}_{ADG})}$ | 0.62(0.07) | 0.62(0.06) | 0.62(0.07) | 0.63(0.07) | 0.63(0.08) |
| ${\rho(TBV}_{ADG},\hat{{TBV}_{ADG})}$ | 0.68(0.06) | 0.69(0.06) | 0.69(0.06) | 0.64(0.06) | 0.63(0.06) |
| ${\rho(DGE}_{BF},\hat{{DGE}_{BF})}$ | 0.69(0.06) | 0.70(0.06) | 0.75(0.06) | 0.74(0.06) | 0.73(0.06) |
| ${\rho(IGE}_{BF},\hat{{IGE}_{BF})}$ | 0.59(0.07) | 0.59(0.07) | 0.59(0.07) | 0.59(0.08) | 0.60(0.07) |
| ${\rho(TBV}_{BF},\hat{{TBV}_{BF})}$ | 0.69(0.06) | 0.69(0.06) | 0.75(0.06) | 0.75(0.06) | 0.73(0.06) |
| $\rho(I_{TBV},\hat{I)}$ | 0.60(0.09) | 0.60(0.1) | 0.50(0.11) | 0.49(0.11) | 0.51(0.12) |
| W_ADG_ – W_BF_, % | 0 – 100 | 25 – 75 | 50 – 50 | 75 – 25 | 100 – 0 |
| W_DGE_ – W_IGE_, % | 100 – 0 | 100 – 0 | 100 – 0 | 100 – 0 | 100 – 0 |
| $\rho({DGE}_{ADG},\hat{{DGE}_{ADG})}$ | 0.69(0.06) | 0.70(0.06) | 0.69(0.06) | 0.64(0.06) | 0.63(0.06) |
| ${\rho(IGE}_{ADG},\hat{{IGE}_{ADG})}$ | 0.63(0.07) | 0.62(0.07) | 0.61(0.07) | 0.62(0.07) | 0.63(0.07) |
| ${\rho(TBV}_{ADG},\hat{{TBV}_{ADG})}$ | 0.69(0.06) | 0.70(0.06) | 0.69(0.06) | 0.64(0.06) | 0.63(0.06) |
| ${\rho(DGE}_{BF},\hat{{DGE}_{BF})}$ | 0.69(0.06) | 0.69(0.06) | 0.74(0.06) | 0.75(0.05) | 0.74(0.06) |
| ${\rho(IGE}_{BF},\hat{{IGE}_{BF})}$ | 0.59(0.07) | 0.59(0.07) | 0.59(0.07) | 0.59(0.07) | 0.59(0.07) |
| ${\rho(TBV}_{BF},\hat{{TBV}_{BF})}$ | 0.69(0.06) | 0.69(0.06) | 0.74(0.06) | 0.75(0.05) | 0.74(0.06) |
| $\rho(I_{TBV},\hat{I)}$ | 0.55(0.12) | 0.55(0.13) | 0.45(0.14) | 0.39(0.16) | 0.41(0.16) |

^a^ Accuracy in generation five. The data were simulated from samples of the marginal posterior distribution of the social effects animal model and the same model was used for genetic evaluation.

^b^ W_ADG_ – W_BF_: proportion of economic weight assigned to average daily gain (ADG) and (backfat thickness) BF in the selection index, W_DGE_ – W_IGE_: proportion of economic weight assigned to direct (DGE) and indirect (IGE) genetic effects of traits (DGE and IGE) in the selection index.

**Table S3** Posterior mean (posterior SD) of the correlation between true figures for total breeding value, direct and indirect genetic effects, and breeding values predictions obtained with the AM ^a^

| W_ADG_ – W_BF_, % ^b^ | 0 – 100 | 25 – 75 | 50 – 50 | 75 – 25 | 100 – 0 |
| --- | --- | --- | --- | --- | --- |
| $\rho(D{GE}_{ADG},\hat{{EBV}_{ADG})}$ | 0.63(0.08) | 0.64(0.08) | 0.63(0.09) | 0.57(0.09) | 0.58(0.08) |
| $\rho({IGE}_{ADG},\hat{{EBV}_{ADG})}$ | -0.04(0.20) | -0.03(0.20) | 0.05(0.21) | 0.05(0.19) | 0.02(0.18) |
| $\rho({TBV}_{ADG},\hat{{EBV}_{ADG})}$ | 0.56(0.10) | 0.58(0.10) | 0.61(0.11) | 0.55(0.10) | 0.54(0.10) |
| $\rho(D{GE}_{BF},\hat{{EBV}_{BF})}$ | 0.66(0.06) | 0.66(0.07) | 0.72(0.06) | 0.72(0.06) | 0.70(0.06) |
| $\rho({IGE}_{BF},\hat{{EBV}_{BF})}$ | 0.06(0.23) | 0.05(0.23) | 0.05(0.26) | 0.07(0.27) | 0.07(0.26) |
| $\rho({TBV}_{BF},\hat{{EBV}_{BF})}$ | 0.60(0.09) | 0.60(0.08) | 0.66(0.09) | 0.66(0.09) | 0.65(0.09) |

^a^ Accuracy in generation five. The data were simulated from samples of the marginal posterior distribution of the social effects animal model but the classical animal was used for genetic evaluation.

^b^W _ADG_ – W_BF_: proportion of economic weight assigned to average daily gain (ADG) and backfat thickness (BF) in the selection index.

**Table S4** Posterior mean (posterior SD) of the correlation between true and predicted breeding values using AM for data generation and evaluation^a^

| W_ADG_ – W_BF_, % ^b^ | 0 – 100 | 25 – 75 | 50 – 50 | 75 – 25 | 100 – 0 |
| --- | --- | --- | --- | --- | --- |
| $\rho(E{BV}_{ADG},\hat{{EBV}_{ADG})}$ | 0.63(0.07) | 0.63(0.07) | 0.63(0.07) | 0.58(0.07) | 0.58(0.07) |
| $\rho({EBV}_{BF},\hat{{EBV}_{BF})}$ | 0.67(0.07) | 0.68(0.07) | 0.72(0.06) | 0.72(0.07) | 0.72(0.07) |

^a^ Accuracy in generation five. The data were simulated from samples of the marginal posterior distribution of the classical animal model (AM) and the same model was used for genetic evaluation.

^b^ W_ADG_ – W_BF_: proportion of economic weight assigned to average daily gain (ADG) and backfat thickness (BF) in the selection index, W_DGE_ – W_IGE_: proportion of economic weight assigned to the EBV’s of traits in the selection index
